# The clinical efficacy of cefoperazone-sulbactam versus piperacillin-tazobactam in the treatment of severe community-acquired pneumonia

**DOI:** 10.1097/MD.0000000000034284

**Published:** 2023-07-14

**Authors:** Chih-Cheng Lai, Wei-Chih Chen, Li-Kuo Kuo, Yao-Tung Wang, Pin-Kuei Fu, Shih-Chi Ku, Wen-Feng Fang, Chin-Ming Chen, Chih-Yen Tu, Wen-Chien Cheng, Chia-Hung Chen

**Affiliations:** a Department of Internal Medicine, Kaohsiung Veterans General Hospital, Tainan Branch, Tainan, Taiwan; b Department of Medicine, Taipei Veterans General Hospital, Taipei, Taiwan; c Division of Pulmonary and Critical Care Medicine, Department of Internal Medicine, Mackay Memorial Hospital, Taipei, Taiwan; d Division of Pulmonary Medicine, Department of Internal Medicine, Chung Shan Medical University Hospital, Taichung, Taiwan; e Department of Critical Care Medicine, Taichung Veterans General Hospital, Taichung, Taiwan; f Division of Chest Medicine, Department of Internal Medicine, National Taiwan University Hospital, Taipei, Taiwan; g Division of Pulmonary and Critical Care Medicine, Department of Internal Medicine, Kaohsiung Chang Gung Memorial Hospital, Kaohsiung, Taiwan; h Department of Intensive Care Medicine, Chi Mei Medical Center, Tainan, Taiwan; i Division of Pulmonary and Critical Care Medicine, Department of Internal Medicine, China Medical University Hospital, Taichung, Taiwan.

**Keywords:** acquired pneumonia, cefoperazone, piperacillin, severe community, sulbactam, tazobactam

## Abstract

The objective was to compare the clinical efficacy of cefoperazone-sulbactam with piperacillin-tazobactam in the treatment of severe community-acquired pneumonia (SCAP). The retrospective study was conducted from March 1, 2018 to May 30, 2019. Clinical outcomes were compared for patients who received either cefoperazone-sulbactam or piperacillin-tazobactam in the treatment of SCAP. A total of 815 SCAP patients were enrolled. Among them, 343 received cefoperazone-sulbactam, and 472 received piperacillin-tazobactam. Patients who received cefoperazone-sulbactam presented with higher Charlson Comorbidity Index scores. (6.20 ± 2.77 vs 5.72 ± 2.61; *P* = .009). The clinical cure rates and effectiveness for patients receiving cefoperazone-sulbactam and piperacillin-tazobactam were 84.2% versus 80.3% (*P* = .367) and 85.4% versus 83.3% (*P* = .258), respectively. In addition, the overall mortality rate of the cefoperazone-sulbactam group was 16% (n = 55), which was also comparable to the piperacillin-tazobactam group (17.8%, n = 84, *P* = .572). The primary clinical outcomes for patients receiving cefoperazone-sulbactam were superior compared to those receiving piperacillin-tazobactam after adjusting disease severity status. The clinical efficacy of cefoperazone-sulbactam in the treatment of adult patients with SCAP is comparable to that of piperacillin-tazobactam. After adjusting for disease severity, cefoperazone-sulbactam tended to be superior to piperacillin-tazobactam.

## 1. Introduction

Community-acquired pneumonia (CAP), and, in particular, severe CAP (SCAP) are significant causes of morbidity and mortality. In spite of evolving and improving therapeutic strategies,^[1]^ the mortality rate of patients with SCAP remains high, ranging from 17% to more than 50%.^[2,3]^ According to the American Thoracic Society/Infectious Disease Society of America guidelines, SCAP is defined as follows: having ≥ 1 of the major criteria, including invasive mechanical ventilation and/or septic shock; having ≥ 3 of the minor criteria, including: respiratory rate ≥ 30 breaths/minutes, partial pressure of oxygen (PaO2)/fraction of inspired oxygen (FiO2) ≤ 250 mm Hg, multiple lobar infiltrates, confusion, blood urea nitrogen ≥ 20 mg/dL, white blood cell count < 4000 cells/mm^3^, platelet count < 10,0000 cells/mm^3^, temperature < 36°C, and/or hypotension requiring aggressive fluid resuscitation.^[4]^ SCAP patients with respiratory failure and invasive mechanical ventilation, severe sepsis or septic shock, and decompensating comorbidities are at increased risk of death.^[5,6]^

*Streptococcus pneumoniae* remains the most common pathogen reported in CAP and is also frequently a cause of SCAP, which generally results in intensive care unit admission.^[7,8]^ *Haemophilus influenza, Legionella pneumonia*, and *influenza pneumonia* are pathogens that also result in SCAP in the intensive care unit.^[9,10]^ However, about 6% of pathogens outside the core microorganisms of CAP, called the PES (*Pseudomonas aeruginosa, extended-spectrum β-lactamase Enterobacteriaceae*, and *methicillin-resistant Staphylococcus aureus*), can also cause severe illness,^[11]^ and the rate of inadequate use of empiric antibiotics is very high. It is important to identify the risk factors of PES in order to choose appropriate antibiotic treatment. The main components of the PES score are as follows: age > 65 years old, male gender, previous antibiotic usage, presence of chronic respiratory disorder, presence of chronic renal disease, impaired consciousness, and fever.^[12]^ Marayuma et al have indicated an antibiotic strategy for pneumonia based on the risk factors for PES pathogens, rather than the site of pneumonia acquisition, as these factors impact the patient PES score.^[13]^ The use of empiric broad-spectrum antibiotics that cover PES pathogens should be considered to improve patient outcomes, especially in those with a PES score ≥ 5.^[14]^

Cefoperazone-sulbactam is a broad-spectrum antibacterial agent and is active against commonly encountered respiratory pathogens and multidrug-resistant organisms, including *Enterobacteriaceae* members, *P aeruginosa*, other non-glucose-fermenting gram-negative bacilli, and anaerobes.^[15]^ Evidence suggests that cefoperazone-sulbactam is not inferior to cefepime and piperacillin-tazobactam for treating hospital-acquired pneumonia and healthcare-associated pneumonia.^[16,17]^ However, studies regarding the clinical effectiveness of cefoperazone-sulbactam in the management of SCAP are lacking. The current study was conducted to clarify the efficacy and safety of cefoperazone-sulbactam versus piperacillin-tazobactam in the treatment of SCAP.

## 2. Materials and methods

### 2.1. Study design and data collection

This study serves as a multicenter retrospective analysis of the clinical effectiveness and safety of cefoperazone-sulbactam in the management of patients suffering from SCAP. The clinical data were extracted from the BATTLE study, which investigated the efficacy and safety of Brosym (TTY Biopharm Company, Taiwan) in the management of SCAP and nosocomial pneumonia. The study was conducted between March 2018 and May 2019 at 8 medical centers and 1 regional hospital in Taiwan. The subjects enrolled in the study received either cefoperazone-sulbactam or piperacillin-tazobactam in the treatment of SCAP, hospital-acquired pneumonia, and ventilator-associated pneumonia. The data were collected retrospectively from electronic medical records after obtaining approval from local ethics committees or institutional review boards. Data included the baseline characteristics of patients, their underlying diseases, type of bacteria observed, type of antibiotic treatment utilized, and clinical outcomes. Due to the retrospective design, individual patient consent was not indicated. All methods were performed in accordance with the Declaration of Helsinki.

### 2.2. Definitions and outcomes

For the purposes of this study, pneumonia was defined as having 2 or more respiratory symptoms and signs (including cough, fever, hypothermia, purulent sputum, or respiratory secretions) and opacity on a chest radiograph as interpreted by the attending physician.^[18]^ The diagnosis of SCAP was based on the American Thoracic Society and Infectious Disease Society of America guidelines.^[4]^ Both respiratory specimens and blood cultures were sampled prior to the prescription of antibiotics. The primary efficacy endpoint was the clinical cure rate of SCAP in response to the antibiotic treatment. Therefore, the cure rate was defined as the proportion of patients in which the clinical symptoms or signs resolved or improved 7 days after the end of antibiotic treatment without additional management. In contrast, clinical failure was defined as follows: clinical symptoms or signs deteriorated or persisted after 3 to 5 days of antibiotic treatment and required additional antibiotics for management; death due to pneumonia after 3 days of antibiotic treatment; or progression of pneumonia and development of empyema or lung abscess. The indeterminate outcomes included transfers to other hospitals, refusal of further treatment, death due to pneumonia following fewer than 2 days of antibiotic treatment, incomplete antibiotic treatment due to allergy, severe adverse events, or other personal reasons. The secondary outcomes measured included the clinical effective rate, the risk of adverse effects, and in-hospital mortality. The clinical effectiveness was defined as the improvement of clinical symptoms and signs, radiographic opacity, and inflammation profiles, including white blood cell counts, procalcitonin, c-reactive protein levels. In contrast, the clinical ineffectiveness was defined as when any one of the 3 criteria mentioned above was not achieved. The indeterminate effectiveness was defined as when the above 3 criteria could not be assessed.

### 2.3. Statistical analysis

Data were compiled and analyzed using the Statistical Analysis System for Windows (Version 9.4 or higher, SAS Institute, Cary, NC). All continuous variables were reported as means with standard deviation or medians with interquartile range. Differences in continuous variables were compared using independent *t* tests or Kruskal–Wallis tests. Meanwhile, categorical variables were reported as the number of patients and percentages. Differences in categorical variables were examined using the F-test or chi-square test. The difference in the crude relative risk and adjusted relative risk (adjusted for propensity score) of the outcomes between cefoperazone-sulbactam versus piperacillin-tazobactam were calculated. All tests of significance were 2-tailed, and a *P* value of ≤.05 was considered to be statistically significant.

## 3. Results

### 3.1. The demographic data

A total of 815 patients with SCAP were enrolled. The baseline characteristics of enrolled patients are shown in Table 1. Among these patients, 343 received cefoperazone-sulbactam, and 472 received piperacillin-tazobactam. The mean age of patients in the cefoperazone-sulbactam group was 74.78 ± 15.00 years, and 242 (70.6%) were male. The median treatment duration of cefoperazone-sulbactam was 9.08 ± 3.53 days. Similar baseline characteristics were noted across both groups, including data related to age, gender, and duration of antibiotic treatment; however, the Charlson Comorbidity Index was significantly higher in the cefoperazone-sulbactam group compared to the piperacillin-tazobactam group (6.20 ± 2.77 vs 5.72 ± 2.61, respectively; *P* = .009). In contrast, there were no significant differences between the 2 groups in the Pneumonia Severity Index scores or the CURB-65 scores. Additionally, the difference of the microbial identification rate between these 2 groups was not statistically significant (49.7% vs 49.6%, respectively; *P* = .981). In the cefoperazone-sulbactam group, Klebsiella pneumoniae (11.1%) and *P aeruginosa* (6.4%) were the most 2 commonly identified pathogens. Similar findings were observed in the piperacillin-tazobactam group. The 2 most common pathogens identified were *P aeruginosa* (8.6%) and *K pneumonia* (8.2%). *S pneumoniae* was not a commonly identified pathogen in patients with SCAP in the current study (4.8%). Regarding adverse events, only 21 patients taking cefoperazone-sulbactam had received an international normalized ratio (INR) test pre- or post-therapy after 1 week. There was no significant difference between the pre-and post-therapy INR in those patients (1.14 vs 1.13; *P* = .597).

**Table 1:** Baseline characteristics of patients with SCAP.

	Cefoperazone-sulbactam N = 343	Piperacillin-tazobactam N = 472	*P* value
Age (yr) Mean ± SD	74.78 ± 15.00	75.14 ± 14.20	.874
Gender, Male (%)	242 (70.6%)	339 (71.8%)	.696
Treatment d Mean ± SD	9.08 ± 3.53	9.31 ± 3.75	.462
Pathogens, n (%)			
*Escherichia coli*	18 (5.2%)	34 (7.2%)	.325
*Klebsiella pneumoniae*	38 (11.1%)	39 (8.2%)	.216
*Pseudomonas aeruginosa*	22 (6.4%)	41 (8.6%)	.286
Acinetobacter spp.	20 (5.8%)	21 (4.4%)	.466
Streptococcus	19 (5.5%)	20 (4.2%)	.487
Other pathogens	50 (14.5%)	74 (15.6%)	.739
No growth/NA	176 (51.3%)	243 (51.4%)	.981
Charlson score, Mean ± SD	6.20 ± 2.77	5.72 ± 2.61	.009*
PSI score, Mean ± SD	146.89 ± 45.34	146.63 ± 43.08	.651
Curb-65 score, Mean ± SD	2.65 ± 1.23	2.60 ± 1.30	.649
In-hospital mortality, n (%)	55 (16.0%)	84 (17.8%)	.572
Primary outcome, n (%)			.367
Clinical cure	287 (84.2%)	378 (80.3%)	
Failure/Indeterminate	54 (15.7%)	93 (19.7%)	
Secondary outcome, n (%)			.258
Effective	293 (85.4%)	393 (83.3%)	
Ineffective/Indeterminate	50 (14.5%)	79(16.7%)	
INR before medicine, n	29	11	.296
Median (95% CI)	1.14 (1.07, 1.19)	1.10 (0.90, 1.82)	
INR after medicine, n	21	6	.579
Median (95% CI)	1.13 (1.09, 1.25)	1.06 (0.94, 1.33)	

CI = confidence interval, INR = international normalized ratio, PSI = pneumonia severity index, SCAP = severe community acquired pneumonia, SD = standard deviation.

* *P* < 0.05.

### 3.2. The clinical characteristics of patients with and without PES

PES pathogens were identified in 8.7% (71/815) of patients with SCAP in our cohort (Table 2). Many of the baseline characteristics in both groups were similar, including age, gender, and underlying comorbidities; however, the prevalence of chronic pulmonary disease was higher in the PES group (36.6% vs 25.8%, *P* = .049). There were no significant differences in the Charlson Comorbidity Index, APACHE II scores, Sequential Organ Failure Assessment (SOFA) scores, and Quick SOFA (qSOFA) scores when comparing the PES and non-PES groups. In spite of these circumstances, the clinical cure rate of patients in the PES group was significantly lower than the rate for the non-PES group (73.2% vs 82.9%, *P* = .021). The in-hospital mortality rate for patients in the PES group was higher than it was for those in in non-PES group (26.8% vs 16.1%, *P* = .023).

**Table 2:** Baseline characteristics of patients with PES or non/unknown PES group.

	PES group	Non-PES or unknown group	*P* value
	N = 71	N = 744	
Age (yr) Mean ± SD	72.46 ± 14.02	75.23 ± 14.57	.073
Gender, Male, n (%)	51 (71.8%)	530 (71.2%)	.916
Myocardial infarction, n (%)	2 (2.8%)	54 (7.3%)	.158
Congestive heart failure, n (%)	8 (11.3%)	107 (14.4%)	.471
Peripheral vascular disease, n (%)	2 (2.8%)	19 (2.6%)	.894
CVA, n (%)	5 (7.0%)	77 (10.3%)	.376
Dementia, n (%)	12 (16.9%)	134 (18.0%)	.816
Chronic pulmonary disease, n (%)	26 (36.6%)	192 (25.8%)	.049*
Connective tissue disease, n (%)	4 (5.6%)	38 (5.1%)	.848
Peptic ulcer disease, n (%)	7 (9.9%)	70 (9.4%)	.901
Liver disease, n (%)	3 (4.2%)	50 (6.7%)	.415
Diabetes mellitus, n (%)	22 (31.0%)	263 (35.3%)	.461
Hemiplegia, n (%)	10 (14.1%)	101 (13.6%)	.905
Moderate to severe CKD, n (%)	9 (12.7%)	124 (16.7%)	.385
Solid tumor, n (%)	7 (9.9%)	69 (9.3%)	.871
Leukemia, n (%)	0 (0.0%)	7 (0.9%)	.412
Lymphoma, n (%)	0 (0.0%)	1 (0.1%)	.757
AIDS, n (%)	0 (0.0%)	3 (0.4%)	.592
Charlson score	5.61 ± 2.70	5.95 ± 2.69	.264
APACHE II score	20.63 ± 8.79	22.15 ± 7.05	.250
SOFA score	6.24 ± 3.80	7.02 ± 3.75	.344
qSOFA score	1.92 ± 0.51	1.74 ± 0.74	.459
Treatment duration (d)	10.25 ± 4.84	9.11 ± 3.51	.108
Primary outcome, n (%)			.021*
Clinical cure	51 (71.8%)	614 (82.9%)	
Failure/Indeterminate	20 (28.2%)	127 (17.1%)	
Secondary outcome, n (%)			.200
Effective	56 (78.9%)	630 (84.7%)	
Ineffective/Indeterminate	15 (21.1%)	114 (15.3%)	
In-hospital mortality, n (%)	19 (26.8%)	120 (16.1%)	.023*

AIDS: = acquired immune deficiency syndrome, CKD = chronic kidney diseases, CVA = cerebral vascular accident, PES = *Pseudomonas aeruginosa*, extended-spectrum β-lactamase Enterobacteriaceae, and methicillin-resistant *Staphylococcus aureus*.

* *P* < 0.05.

### 3.3. Treatment outcomes

**Figure 1.**
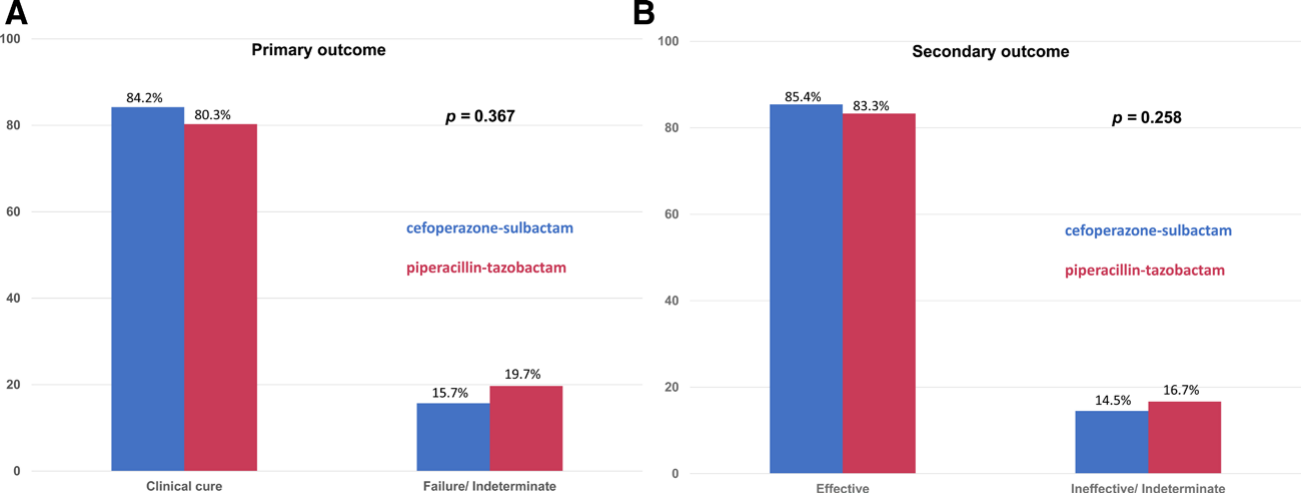
(A) Primary outcome and (B) secondary outcome of patients receiving cefoperazone-sulbactam and piperacillin-tazobactam for severe community - acquired pneumonia.

**Figure 2.**
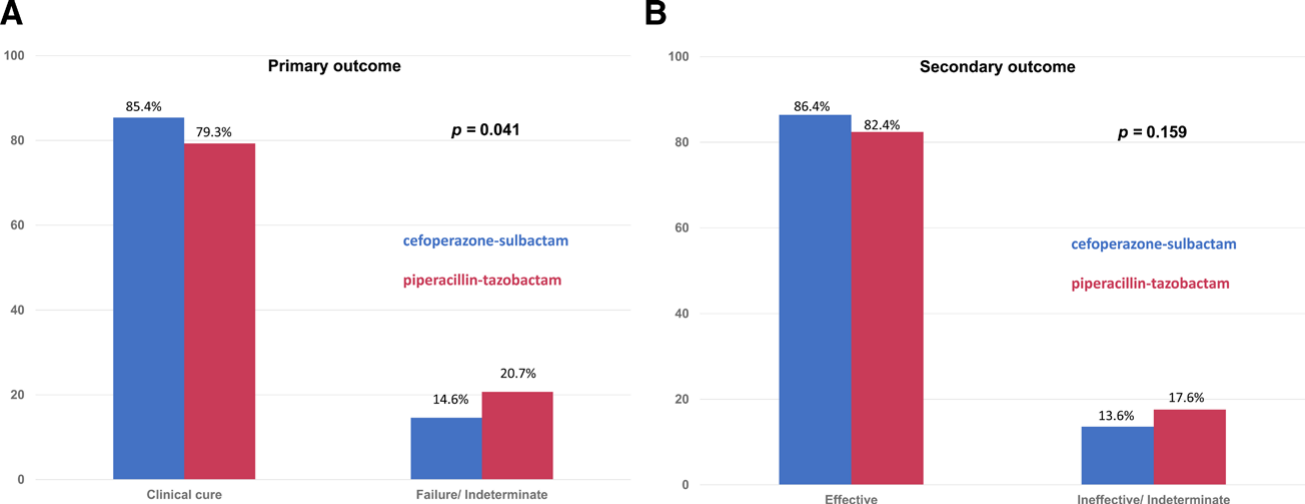
(A) Primary outcome and (B) secondary outcome of patients receiving cefoperazone-sulbactam and piperacillin-tazobactam for severe community - acquired pneumonia after adjusting propensity score.

The primary outcomes of clinical cure rate and failure (or indeterminate) rate between the cefoperazone-sulbactam and piperacillin-tazobactam groups were 84.2% vs 80.3% and 15.7% vs 19.7%, respectively (*P* = .367) (Fig. 1A). For the secondary outcomes, the clinical effectiveness rate and ineffective (or indeterminate) rates of cefoperazone-sulbactam were 85.4% and 14.5%, which were similar to rates for the piperacillin-tazobactam group (83.3% and 16.7%; *P* = .258) (Fig. 1B). Furthermore, the in-hospital mortality rate was similar between the cefoperazone-sulbactam and piperacillin-tazobactam groups (16.0% vs 17.8%; *P* = .572) (Table 1). On the other hand, based off of the Charlson scores, disease severity was different between the 2 groups. The propensity score method was performed to adjust for this confounding factor by adjusting the Charlson score (see Table, http://links.lww.com/MD/J266, Supplemental Content, which illustrates the baseline characteristics after the propensity score matching between these 2 groups). The clinical cure rate of cefoperazone-sulbactam was higher than the cure rate with piperacillin-tazobactam (85.4% vs 79.3%; *P* = .041) (adjusted odds ratio, 1.53; 95% confidence interval, 1.02–2.31). No significant difference was found between the 2 groups in terms of secondary outcomes or in-hospital mortality (Fig. 2 and Table 3).

**Table 3:** Outcome analysis of patients receiving cefoperazone-sulbactam versus piperacillin-tazobactam.

N = 324	*Primary outcome*		*Secondary outcome*		*In-hospital mortality*	
	Crude OR (95% CI)	Adjust OR* (95% CI)	Crude OR (95% CI)	Adjust OR* (95% CI)	Crude OR (95% CI)	Adjust OR* (95% CI)
Reference = piperacillin-tazobactam						
Cefoperazone-sulbactam	1.53 (1.02, 2.31)	1.53 (1.02, 2.31)	1.36 (0.89, 2.08)	1.36 (0.89, 2.08)	1.34 (0.90, 2.01)	1.34 (0.90, 2.01)

CI = confidence interval, OR = odds ratio.

* Adjust OR by Wald method.

## 4. Discussion

The major findings in the current study can be summarized as follows: First, for the treatment of SCAP, the clinical cure rates and effectiveness rates of cefoperazone-sulbactam were similar to those of piperacillin-tazobactam; however, cefoperazone-sulbactam demonstrated superior clinical primary outcomes following adjustment of Charlson scores. The in-hospital mortality was similar between patients receiving either of these 2 antibiotics. Finally, PES pathogens were more commonly identified in patients with chronic pulmonary disease, and these patients tended to experience poor outcomes.

The hospital mortality of SCAP is still high, ranging from 17% to over 50%.^[2,3]^ Because of the emergence of pathogens outside of the core microorganisms of CAP,^[12]^ it is crucial to administer adequate broad-spectrum antibiotics covering PES pathogens in the early stages of treatment.^[19]^ In this study, PES pathogens were identified in 8.7% of SCAP patients, which is consistent with several previous studies, which identified these pathogens in 6% to 7% of CAP patients.^[12,20]^ In the current study, *P aeruginosa* (7.7%) and *K pneumoniae* (9.3%) were the most 2 commonly identified pathogens resulting in SCAP. These were followed by *Escherichia coli* (6.3%), *Acinetobacter spp.* (5.0%), and *S pneumoniae* (4.7%). The hospital mortality rates for SCAP patients receiving cefoperazone-sulbactam and piperacillin-tazobactam were 16.0% and 17.8%, respectively; however, the hospital mortality rate of SCAP patients with PES pathogens was higher than the rate for those with non-PES pathogens (26.8% vs 16.1%, *P* = .023). In our cohort, the mortality rate was lower in patients suffering from SCAP compared to previous reports, which suggests that broad-spectrum antibiotics should be used initially.^[19]^

Risk factors associated with increased chance of death have been identified, including patient indication of prior antibiotic usage, use of proton pump inhibitor medications, need for tube feeding, non-ambulatory status,^[20]^ CAP severity, recent hospitalization, dialysis, and immunosuppression.^[13]^ Furthermore, male sex, presence of chronic airway disease, need for vasoactive drug or mechanical ventilation, higher pneumonia severity index, and prior *P aeruginosa* colonization have been identified as risk factors for *P aeruginosa* CAP.^[11]^ Chronic pulmonary disease was the only risk factor identified for the SCAP patients with PES pathogens in our cohort, which is consistent with the previous report.^[11]^

Cefoperazone is effective not only against gram-positive aerobes but also against a wide variety of gram-negative aerobes, including *Enterobacteriaceae* members, *P aeruginosa*, and other non-glucose-fermenting Gram-negative bacilli in vitro.^[21]^ However, its minimum inhibitory concentration is influenced by the high inoculum concentration of B-lactamase-producing organisms.^[21]^ When combined with sulbactam, the bacterial inoculum effect against cefoperazone can be overcome^[22]^ and can increase the additional coverage of anaerobes.^[23]^ In addition, sulbactam contains intrinsic antibacterial activity to combat *Acinetobacter species*.^[24]^ Jean SS et al reported the susceptibility rates of *E coli, K pneumoniae, Citrobacter freundii, Serratia marcescens, Proteus mirabilis, P aeruginosa, Enterobacter cloacae*, and *Acinetobacter spp.* to cefoperazone-sulbactam were all above 85%.^[25]^ For drug resistant organisms, the susceptibility rates to

cefoperazone-sulbactam have been shown to be 97% (ESBL-*E coli*), 75.8% (ESBL-*K pneumoniae*), 67.6% (ESBL-*K pneumoniae*), and 68% (carbapenem-resistant *A. baumannii*).^[22]^ Piperacillin-tazobactam was the most commonly prescribed antibiotic for suspected drug resistant pathogens, but it has been shown to have adverse outcomes in patients with *E coli* and *K pneumoniae* bacteremia.^[26]^ The current study demonstrated a superior clinical cure rate with cefoperazone-sulbactam compared to piperacillin-tazobactam after adjustment severity scores for SCAP. Based on the research indicated above, cefoperazone-sulbactam has been shown to be another alternative antibiotic to utilize in the fight against antibiotic-resistant bacteria.

On the other hand, there are safety considerations to be taken into account when prescribing cefoperazone-sulbactam. The coagulopathy, due to its N-methylthiotetrazole side chain, potentially induces transient hypoprothrombinemia.^[27]^ This complication is generally rare and is only observed in patients concurrently receiving anticoagulants, in malnourished patients, and in patients who have experienced hemorrhagic events in the recent half-year.^[28]^ In the current study, no significant difference in the pre-and post-INR in was observed with the 21 patients receiving cefoperazone; however, most of the patients were not routinely assessed for bleeding disorders because they did not present with significant bleeding in our retrospective study.

We acknowledge some limitations to this study. First, the retrospective design inherently cannot ensure baseline equality between the 2 groups. The cefoperazone-sulbactam group displayed a significantly higher Charlson Comorbidity Index compared to the piperacillin-tazobactam group, making it difficult to provide a clear explanation for this deviation. Although there is no issue of overlapping between these 2 groups, the magnitude of potential bias remains uncertain. As a result, the propensity score matching method was performed to adjust the baseline clinical characteristics. In the future, it is advisable to conduct a prospective randomized controlled study to mitigate potential bias. Secondly, the PES score was not reported for each patient; however, the severity of CAP was high enough to prescribe broad-spectrum antibiotics. The identification of PES pathogens may have been underestimated, as the microbial identification rate between the 2 groups was around 50%. Lastly, mild adverse events were not recorded, and some patients did not receive INR measurement. Therefore, the risk of adverse events and the safety of cefoperazone-sulbactam could have been underestimated.

In summary, this study suggests cefoperazone-sulbactam is as effective as piperacillin-tazobactam in the treatment of patients with SCAP. After adjusting for disease severity, the clinical cure rate of cefoperazone-sulbactam tended to be superior to piperacillin-tazobactam in treating SCAP.

## Author contributions

**Conceptualization:** Wen-Chien Cheng, Chih-Cheng Lai, Chih-Yen Tu, Chia-Hung Chen.

**Data curation:** Chih-Cheng Lai, Wei-Chih Chen, Li-Kuo Kuo, Yao-Tung Wang, Pin-Kuei Fu, Shih-Chi Ku, Wen-Feng Fang, Chin-Ming Chen, Chih-Yen Tu, Chia-Hung Chen.

**Formal analysis:** Chia-Hung Chen.

**Funding acquisition:** Chih-Yen Tu.

**Investigation:** Chih-Yen Tu.

**Methodology:** Chih-Cheng Lai, Chih-Yen Tu.

**Project administration:** Chih-Yen Tu.

**Resources:** Chih-Yen Tu.

**Software:** Chih-Yen Tu.

**Supervision:** Pin-Kuei Fu, Shih-Chi Ku, Wen-Feng Fang, Chin-Ming Chen, Chih-Yen Tu, Chia-Hung Chen.

**Validation:** Wei-Chih Chen, Li-Kuo Kuo, Yao-Tung Wang, Pin-Kuei Fu, Shih-Chi Ku, Wen-Feng Fang, Chin-Ming Chen, Chih-Yen Tu, Chia-Hung Chen.

**Visualization:** Yao-Tung Wang, Chih-Yen Tu, Chia-Hung Chen.

**Writing – original draft:** Wen-Chien Cheng, Chih-Cheng Lai, Chia-Hung Chen.

**Writing – review & editing:** Wen-Chien Cheng, Chih-Cheng Lai, Chia-Hung Chen.

## Supplementary Material


