# Supplementary material for: The clinical efficacy of cefoperazone-sulbactam versus piperacillin-tazobactam in the treatment of severe community-acquired pneumonia
Source: Medicine (Baltimore). 2023 Jul 14;102(28):e34284. doi: 10.1097/MD.0000000000034284 (PMC10344575; doi:10.1097/MD.0000000000034284)
Supplement: Supplementary file 1 [file medi-102-e34284-s001.pdf]

Table supplement. Baseline characteristics of patients with SCAP by propensity score matching using Charlson score

	cefoperazone-sulbactam N=324	piperacillin-tazobactam N=324	*p*-value
**Charlson score, Mean ± SD**	6.06 ± 2.59	6.06 ± 2.59	>0.999
**Gender, Male (%)**	226 (69.8%)	228 (70.4%)	0.864
**Myocardial infarction, n (%)**	23 (7.1%)	21 (6.5%)	0.755
**Congestive heart failure, n (%)**	44 (13.6%)	53 (16.4%)	0.322
**Peripheral vascular disease, n (%)**	6 (1.9%)	12 (3.7%)	0.151
**CVA, n (%)**	40 (12.3%)	31 (9.6%)	0.258
**Dementia, n (%)**	75 (23.1%)	48 (14.8%)	0.007*
**Chronic pulmonary disease, n (%)**	87 (26.9%)	89 (27.5%)	0.860
**Connective tissue disease, n (%)**	24 (7.4%)	14 (4.3%)	0.095
**Peptic ulcer disease, n (%)**	32 (9.9%)	33 (10.2%)	0.896
**Liver disease, n (%)**	3 (4.2%)	50 (6.7%)	0.415
**Hemiplegia, n (%)**	61 (18.8%)	34 (10.5%)	0.003*
**Moderate to severe CKD, n (%)**	53 (16.4%)	60 (18.5%)	0.469
**Solid tumor, n (%)**	31 (9.6%)	28 (8.6%)	0.682
**Leukemia, n (%)**	4 (1.2%)	0 (0.0%)	0.045*
**AIDS, n (%)**	1 (0.3%)	1 (0.3%)	>0.999
**Overall Mortality, n (%)**	51 (15.7%)	65 (20.1%)	0.151
**Primary outcome, n (%)**			
Clinical cure	277 (85.4%)	257 (79.3%)	0.041*
Failure/ Indeterminate	47 (14.6%)	67 (20.7%)	
**Secondary outcome, n (%)**			
Effective	280 (86.4%)	267 (82.4%)	0.159
Ineffective/ Indeterminate	44 (13.6%)	57 (17.6%)	

AIDS: acquired immune deficiency syndrome; CVA: Cerebrovascular accident; SD: standard deviation
